# Efficacy of Maitland Mobilization and Myofascial Release as Preoperative Care in Ankle Arthritis With Severe Equinus Deformity: A Rare Case Report

**DOI:** 10.7759/cureus.65979

**Published:** 2024-08-01

**Authors:** Pooja R Tiwari, Sandeep Shrivastav, Mitushi Deshmukh, Nikita Kaple

**Affiliations:** 1 Department of Musculoskeletal Physiotherapy, Ravi Nair Physiotherapy College, Datta Meghe Institute of Higher Education and Research (Deemed to be University), Wardha, IND; 2 Department of Orthopaedics, Datta Meghe Institute of Higher Education and Research (Deemed to be University), Wardha, IND

**Keywords:** post-traumatic ankle joint osteoarthritis, physiotherapy, pain, ankle arthritis, ankle joint

## Abstract

Advanced post-traumatic ankle osteoarthritis (PTAO) is a severe condition that affects less than one percent of the population, with rare incidence. It accounts for less than 5% of all osteoarthritis (OA) cases. Physiotherapy enhances functionality by strengthening the dynamic stabilizers of the ankle, such as the calf, soleus, tibialis anterior, and peroneal muscles, and by improving proprioception, which aids in balance and coordination. As OA progresses, individuals may experience early losses in their ability to perform everyday activities and job tasks. Occupational therapy and cardiovascular exercises are crucial for conserving energy while walking and improving posture at work. This case report involves a 39-year-old male who presented to the hospital with pain, swelling, difficulty walking, and an equinus deformity. After diagnosing him with ankle arthritis, the orthopedic specialist recommended an X-ray. Medication and physical therapy were administered to educate and rehabilitate the patient, aiming to improve pain, range of motion (ROM), strength, and walking capacity. A four-week treatment plan, along with medication, resulted in significant improvements in pain reduction, ROM, strength, and walking ability. This case report also underscores the importance of focusing on preoperative care to ensure that post-surgery, the hip and knee ranges are normal, and the patient experiences less difficulty walking. Future studies are needed to explore this condition further and to evaluate the effectiveness of ultrasound therapy in such cases, as it was not effective in reducing pain in this instance.

## Introduction

The ankle joint is a complicated structure composed of a synovial joint between the tibia and talus (talocrural) and a fibrous syndesmosis between the tibia and fibula. The medial (deltoid) and lateral collateral ligaments provide stability and enable hinge-like movements, such as dorsiflexion and plantarflexion, as well as slight rotation and inversion/eversion. Compared to the hip and knee joints, the ankle's range of motion (ROM) during walking is relatively limited, often reaching up to 30°, but expanding to around 56° when descending stairs [1]. Ankle fractures are frequent and widespread injuries. Indeed, fractures involving the malleoli account for approximately 9-18% of all fractures seen in the emergency department. These fractures can present in several forms, such as those involving only the fibula, only the tibia, both malleoli (bimalleolar), and all three malleoli (trimalleolar), and may also include other associated injuries [2]. Surgical intervention is generally the preferred treatment for ankle fractures, except for elderly individuals with poorly managed diabetes, active chronic inflammatory illnesses, extreme edema, obesity, or peripheral vascular problems, as well as some non-displaced fractures. Malunions can occur due to fracture complexity, insufficient alignment post-reduction, or further alignment loss following surgery. These malunions may affect joint alignment, articular surface congruity, and the distribution of physiological stresses inside the joint [3]. Failure to address these conditions invariably increases the risk of developing post-traumatic ankle osteoarthritis (PTAO).

Despite advancements in treatment approaches and the implementation of optimal surgery to achieve anatomically precise and stable fracture reduction, the long-term risk of PTAO remains notably high, ranging between 20% and 40% [4]. Poliomyelitis, meningomyelocele, arthrogryposis multiplex congenital, and post-traumatic diseases can all cause significant foot and ankle deformities in adults. Osteotomies and/or progressive correction can treat these severe anomalies with an external fixator. Some people in developing countries may not choose this option due to the cost and time involved in gradual deformity correction; therefore, single-stage deformity treatment may be a better option for them. The literature mentions tibia-calcaneal fusion in adults as a treatment for post-traumatic avascular necrosis of the talus and neuropathic ankle deformity [5].

Advanced PTAO is a serious condition that affects roughly 1% of the general population. Certain studies suggest an incidence rate of around 30 cases per 100,000 people, accounting for approximately 2-4% of total osteoarthritis (OA) occurrences [6]. The number of cases of PTAO is increasing, resulting in severe social and economic difficulties. This is particularly significant because PTAO frequently affects younger individuals compared to OA affecting other joints [7]. The onset of PTAO following an ankle fracture might be impacted by both known and unforeseen circumstances. Trauma severity appears as a crucial predictor of PTAO development. While the prevalence of PTAO post-fracture appears to be common regardless of fracture form, specific fractures provide a larger risk. Fractures caused by exposure or dislocation are particularly connected to an increased risk of early PTAO. Despite extensive efforts, orthopedic surgeons can mitigate but not eliminate the risk of PTAO. 

Patients should also be informed about potential treatment options for PTAO. For mild to moderate arthritis, regenerative therapy can be considered, as well as osteophyte excision if necessary. In situations with advanced PTAO, treatments such as ankle fusion or total ankle arthroplasty (TAA) with cutting-edge technologies remain the preferred treatment approaches [8]. Ankle arthritis arises from the breakdown of cartilage within the ankle joint, often stemming from various causes such as trauma (e.g., car accidents), autoimmune disorders (e.g., rheumatoid arthritis), or infection. Most commonly, ankle arthritis results from cartilage degeneration due to prior injury. Symptoms typically encompass pain aggravated by activity, stiffness or reduced mobility, swelling, and ankle deformity. Diagnosis typically involves X-rays, CT scans, and MRI imaging. Treatment approaches range from non-surgical to surgical interventions. Non-surgical methods may include wearing ankle braces, cortisone injections, modifying activities to avoid high-impact movements, and utilizing ice and anti-inflammatory medications. Surgical options encompass ankle fusion (arthrodesis), total ankle joint replacement (arthroplasty), and bone spur removal (debridement) [9]. Physical therapy endeavors to enhance functionality by targeting the strengthening of dynamic stabilizers in the ankle, including the calf, soleus, tibialis anterior, and peroneal muscles. In addition, it focuses on improving proprioception, which is the joint's awareness of its position in space, thereby aiding in better balance and coordination [10]. As OA progresses, patients could experience early decreases in their ability to do daily activities and decreased performance at work. In response, occupational therapy and aerobic exercise play critical roles in conserving energy while walking and optimizing posture during work duties. Furthermore, therapeutic methods such as electrical stimulation, thermotherapy, electrotherapy, or ultrasound may be applied to reduce symptoms and give relief [11].

## Case presentation

**Figure 1:**
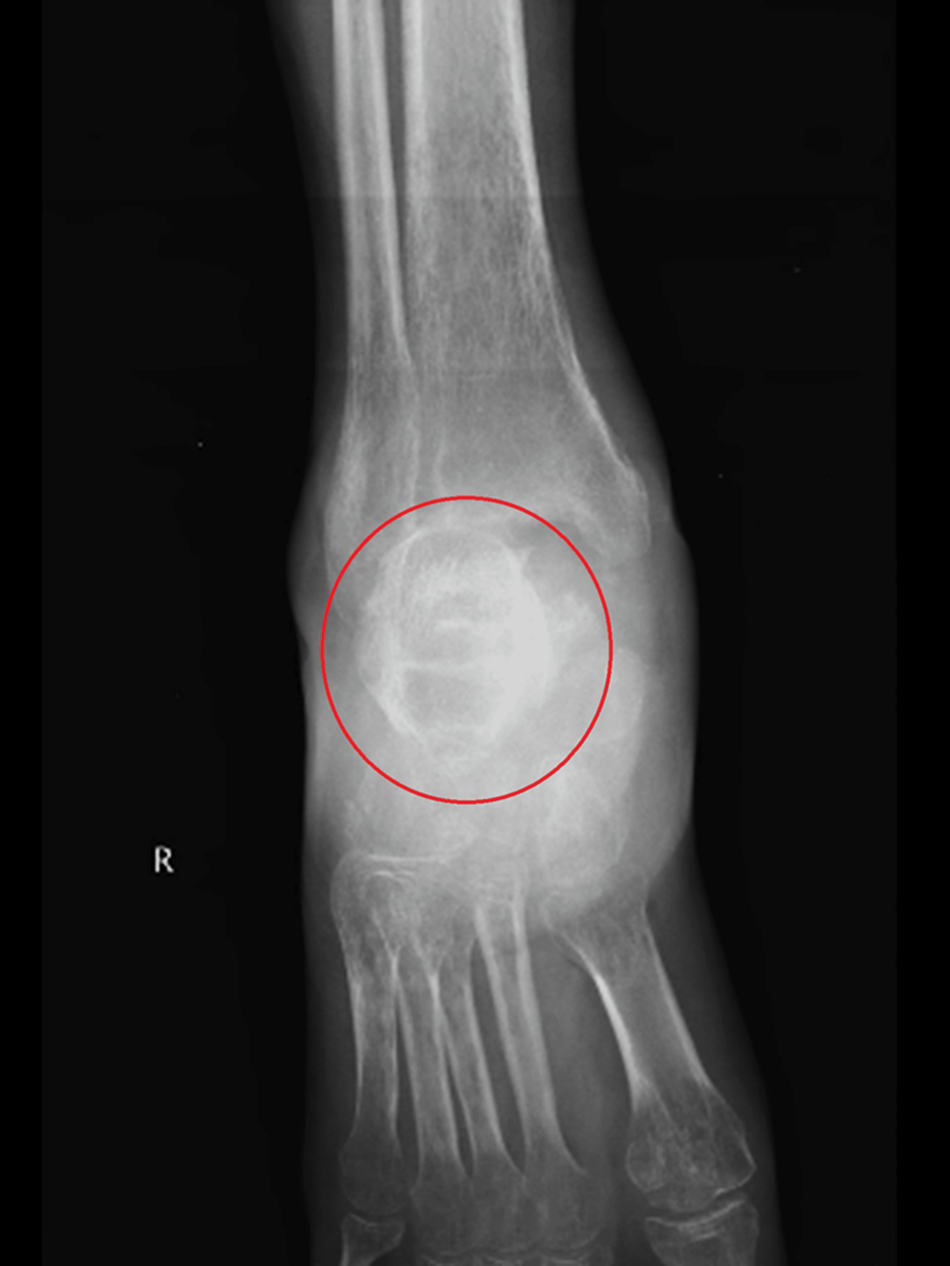
X-ray of the right ankle joint from the frontal view showing the arthritic changes of the talus bone.

**Figure 2:**
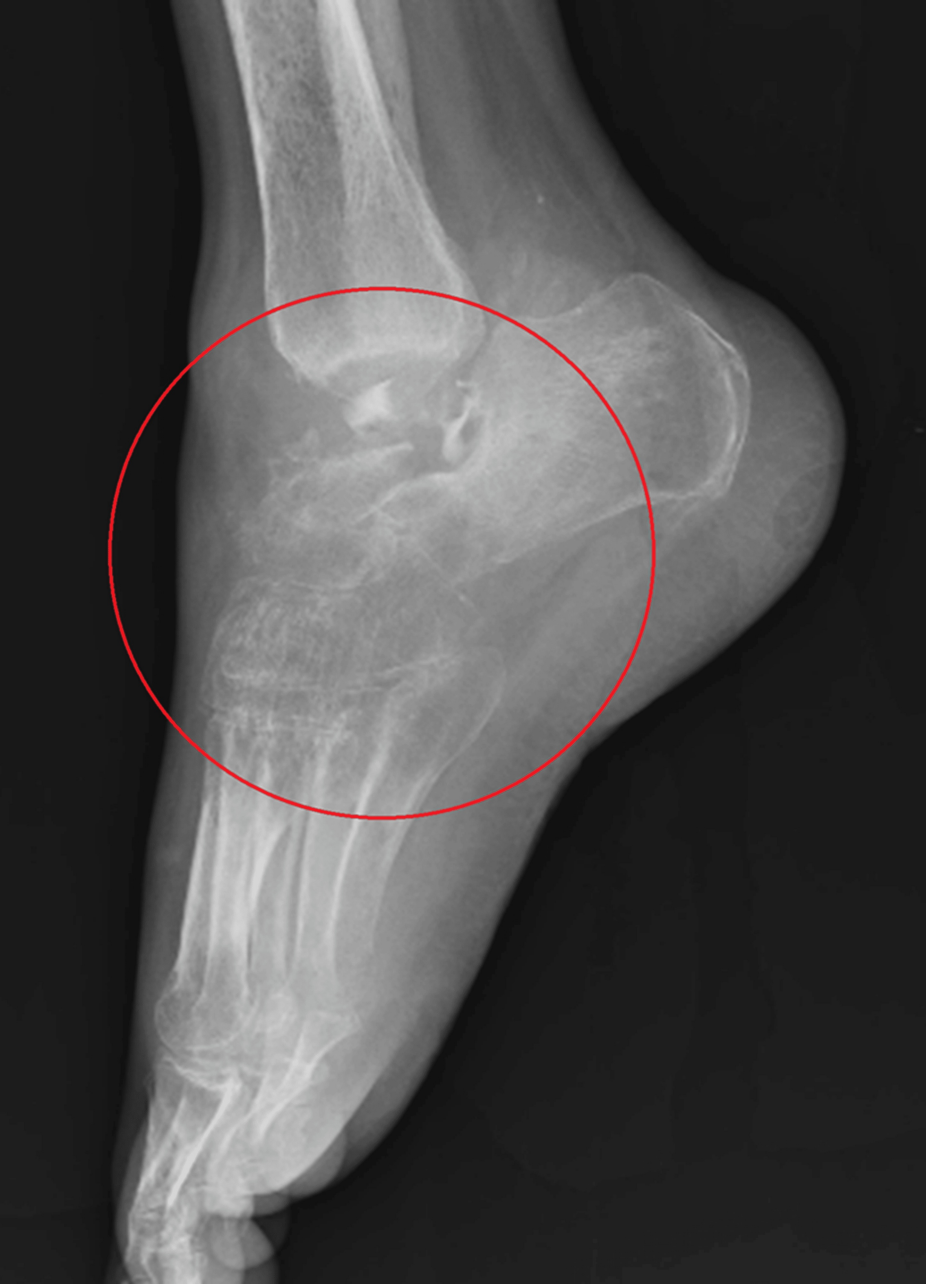
X-ray of the right ankle taken from the lateral view showing arthritic changes.

The patient in this case is a 39-year-old male from Khadaki, Wardha, who has been complaining of pain in his right ankle for the past 1.5 years and has had trouble walking during that time. One year ago, the patient was fine, but then he suffered a fracture of his right talus, for which he underwent surgical treatment. The pain began gradually and has been increasing, reaching a level of 7/10 on the numerical pain rating scale (NPRS). It is characterized by a dull aching pain that worsens with movement and exertion but is sometimes eased by rest and medication. Over six months, his pain worsened to the point that it greatly hampered his daily tasks, such as walking and squatting, making his occupation as a farmer difficult; along with this, he also suffered from equinus deformity. Despite seeking relief, the pain persisted, prompting the patient to visit the Orthopaedic Department of Acharya Vinoba Bhave Rural Hospital (AVBRH) in May 2024, citing pain in his right ankle and difficulty walking. An X-ray was performed as recommended by the consulting orthopedic surgeon, revealing arthritic changes in his ankle joint, as shown in Figures 1-2. Subsequently, physiotherapy was initiated to manage pain and prevent further complications.

Clinical findings

**Figure 3:**
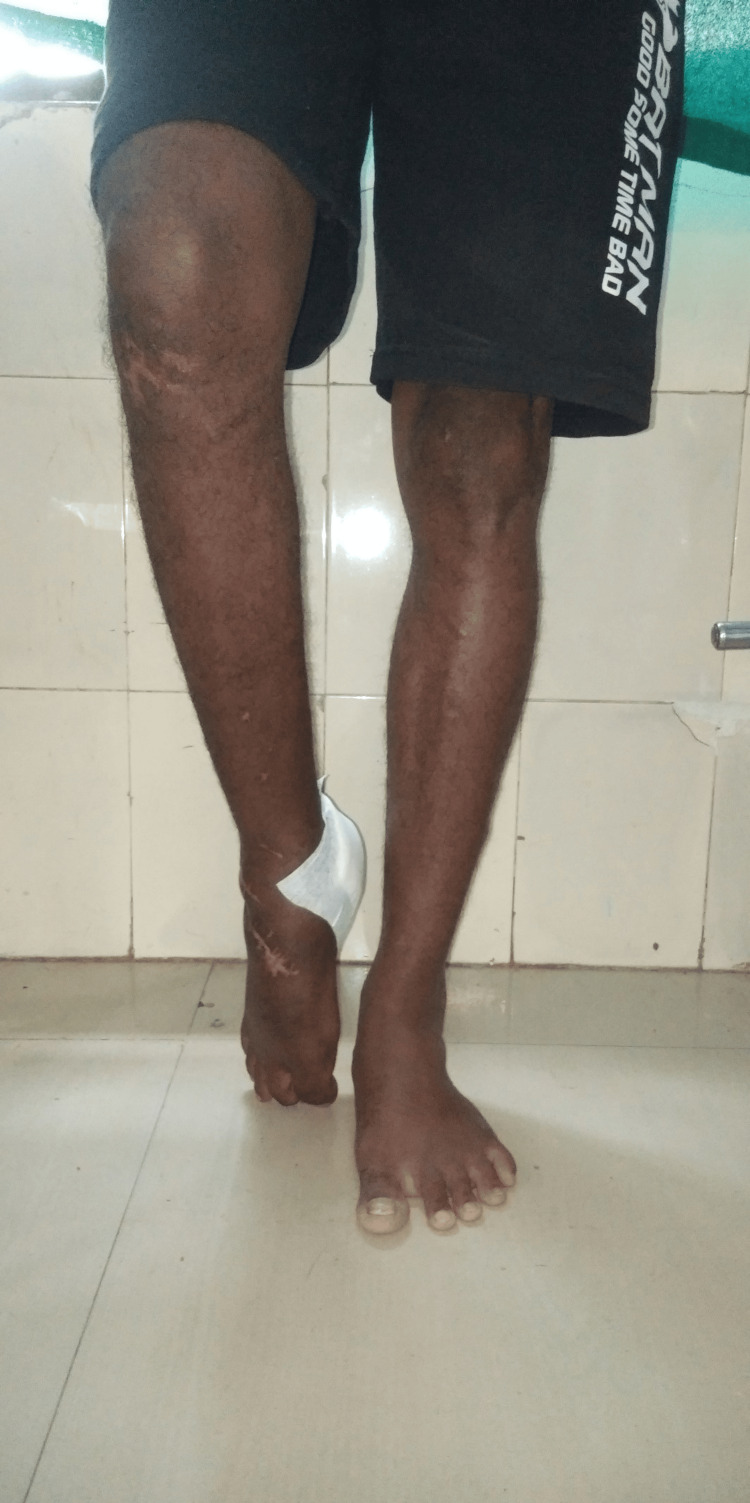
Equinus deformity on the right leg.

The patient was assessed in a supine position. Upon general examination, the patient's vital signs were within normal range: afebrile, with a pulse rate of 88 beats per minute, a respiratory rate of 20 breaths per minute, a blood pressure of 126/80 mmHg, and a BMI of 24.9 kg/m^2^. The underlying skin appeared normal, although a scar from a previous operation was evident upon examination. The patient demonstrated 40 degrees of plantar flexion in the right lower limb, as shown in Figure 3. Local temperature was elevated upon examination of the right ankle, with grade 2 tenderness noted over the right ankle joint. The ROM of the right lower limb is detailed in Table 1. Manual muscle testing of the right ankle joint is provided in Table 2. The gastrocnemius, soleus, and plantaris muscles on the affected side were tight. Normal reflexes and sensory testing were observed during the neurological examination on both sides. Muscle weakness was noted in the right lower limb compared to the left. The patient exhibited an altered gait pattern, specifically toe walking.

**Table 1:** Lower limb range of motion.

Lower limb range of motion	Pre– interventional right	Post– interventional right	Pre– interventional left	Post– Interventional left
Hip flexion	110˚	120˚	120˚	110˚
Hip extension	20˚	30˚	30˚	20˚
Hip abduction	40˚	45˚	40˚	40˚
Hip adduction	30˚	30˚	30˚	30˚
Hip internal rotation	40˚	45˚	40˚	45˚
Hip external rotation	40˚	45˚	40˚	45˚
Knee flexion	110˚	120˚	130˚	130˚
Knee extension	110-15˚	110-10˚	130-0˚	130-0˚
Ankle plantarflexion	40˚	50˚	40˚	50˚
Ankle dorsiflexion	0˚	10˚	15˚	20˚
Ankle eversion	5˚	10˚	5˚	10˚
Ankle inversion	10˚	15˚	5˚	10˚

**Table 2:** Lower limb manual muscle testing.

Manual muscle testing for the upper limb	Pre-interventional right	Post– interventional right	Pre– interventional left	Post– Interventional left
Hip flexors	3/5	4/5	4/5	5/5
Hip extensors	3/5	4/5	4/5	5/5
Hip abductors	3/5	4/5	4/5	5/5
Hip adductors	3/5	4/5	4/5	5/5
Hip internal rotators	3/5	4/5	4/5	5/5
Hip external rotators	3/5	4/5	4/5	5/5
Knee flexors	3/5	4/5	4/5	5/5
Knee extensors	2/5	3/5	4/5	5/5
Ankle dorsiflexors	1/5	2/5	4/5	5/5
Ankle Planterflexors	1/5	2/5	4/5	5/5
Ankle evertors	1/5	2/5	4/5	5/5
Ankle invertors	1/5	2/5	4/5	4/5

Physiotherapy protocol

**Figure 4:**
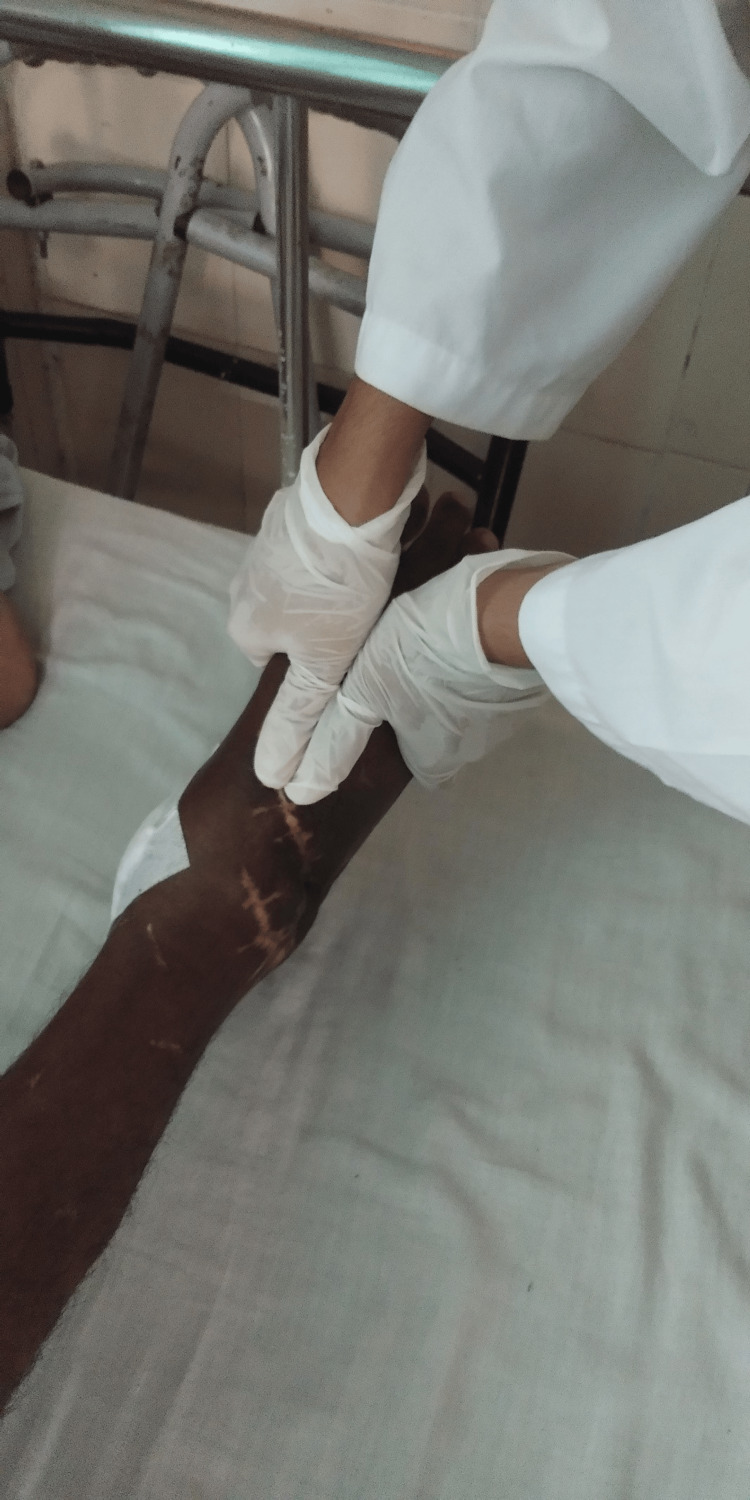
The therapist is giving Maitland mobilization glides to reduce pain and improve the range of motion of the ankle joint.

**Figure 5:**
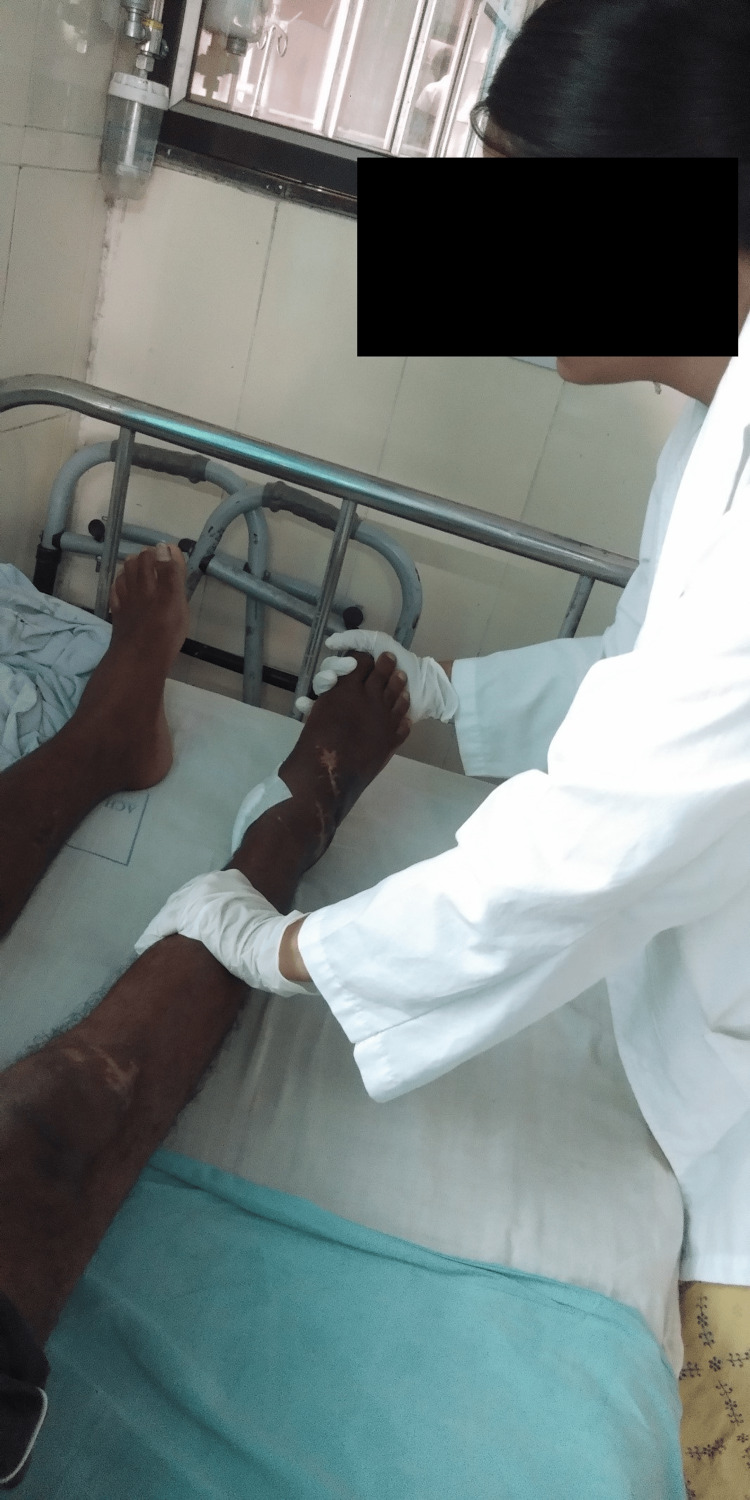
The therapist is administering stretching exercises to the calf muscle.

To cope with his condition, the patient was instructed to adhere to a physiotherapy routine, take medication, and rest appropriately. The objectives were to relieve discomfort, avoid impairment, and enhance his right knee and ankle ROM, as illustrated in Table 3. We also added the manual therapy of Maitland's glides followed by stretching, as described in Figures 4-5. Table 4 shows the outcome measures used to assess the effectiveness of physiotherapy.

**Table 3:** Physiotherapy protocol.

Days/weeks	Regimens	Physiotherapy interventions	Dosage
Week 1	To educate the patient	Patient education and self-management are both advised. The physiotherapist can explain the significance of exercises and how they help prevent further joint damage.	
Week 1	To protect the joint from further damage	Protective bracing can prevent joints from further damage.	8-12 hours a day
Week 1	To relieve pain	Heat therapy in pain relieving for chronic stages.	20-30 minutes twice a day
Week 1	For pain	Ultrasound therapy	1-2 w/cm^2 ^once a day
Week 2	To prevent disability	Maitland mobilization (passive Joint mobilization): This involves movements to enhance plantarflexion, dorsiflexion, inversion, and eversion of the ankle. It has been shown to effectively alleviate pain and improve joint mobility. However, it is only beneficial when combined with active exercise therapy. Anterior and posterior ankle joint glides can also be utilized.	Once a day
Week 2	For disability prevention	Myofascial release (MFR) for Achilles tendon tightness: Identify specific areas of tightness or knots in the Achilles tendon and surrounding calf muscles (gastrocnemius and soleus). Using fingers, knuckles, or elbows, apply gentle but firm pressure to the tight areas. Hold the pressure for several seconds to a few minutes, allowing the tissues to slowly stretch the Achilles tendon and calf muscles while maintaining the pressure. This can be done by dorsiflexing the foot (pulling the toes towards the shin). to soften and release.	Twice a day
Week 2	For disability prevention	Massage techniques: targeting the calf muscles	Once a day
Week 2	To improve strength and endurance	Isometrics "static muscle contractions’ muscle strengthening exercises for gastrocnemius, soleus, tibialis anterior, and peronei.	10 reps × 1 set twice a day
Week 3	To increase functional capacity	ROM exercises: Perform a range of motion exercises, including plantar flexion, dorsiflexion, inversion, and eversion of the ankle. These activities are recommended to improve flexibility and mobility.	10 reps × 1 set twice a day
Week 4	To promote general fitness	Endurance training: Engage in exercises aimed at enhancing aerobic capacity, such as running, stair climbing, and cycling. Functional activities include standing on one leg, walking on different surfaces, sitting and standing up, getting out of laying positions, and ascending stairs. These workouts engage numerous components simultaneously, improving overall functionality.	Twice a day

**Table 4:** Outcome measures.

Outcome measures	Pre-interventional (baseline)	Post-interventional (after four weeks)
Numerical Pain Rating Scale (NPRS)	7/10	3/10
The Intermittent and Constant Osteoarthritis Pain index (ICOAP)	80	40
Dynamic Gait Index	19	24
Foot and Ankle Disability Index	66	100

## Discussion

Ankle arthritis is a prevalent condition associated with substantial socioeconomic impact and morbidity. Patients affected by ankle arthritis are typically divided into two categories that are older people with major comorbidities and young adults who have previously sustained ankle trauma that resulted in post-traumatic arthritis. Management options for ankle arthritis differ greatly due to the large range of patient demographics and clinical manifestations. Non-operative medical care approaches aim to lessen weight-bearing forces at the ankle joint. This includes changing activities, weight reduction, using walking aids, avoiding impact sports, and concentrating on non-weight-bearing exercises like swimming and cycling. Patients could benefit from ankle braces or boots, additionally to footwear modifications such as rocker-soled shoes that restrict ankle joint motion. Before considering surgical intervention, patients are recommended for physiotherapy and clinical interventions, analgesics, and anti-inflammatory drugs [12]. Increased muscular stiffness can reduce ROM and impede function. Reduced ankle dorsiflexion ROM is connected with an increased risk of ankle injury. Although self-myofascial release (SMFR) is widely employed in clinical and sports settings, the effects on gastrocnemius muscle and Achilles tendon stiffness are poorly understood. As a result, we investigated the effects of SMFR with a foam roller (FR) on gastrocnemius-AT complex stiffness and ankle dorsiflexion ROM. The findings indicate that applying an FR to the calf can effectively relax the gastrocnemius muscle and enhance ankle dorsiflexion ROM. Myofascial release (MFR) is a manual therapy that involves gradually stretching the myofascial complex to restore ideal length, relieve pain, and improve function [13].

In March 2024, a study looked at the safety and efficacy of myofascial release therapy for individuals with hemophilic ankle arthropathy. This single-blind, randomized controlled experiment included 58 adults with hemophilia. Participants were randomly assigned to one of two groups: the experimental group, which underwent myofascial release therapy using a foam roller, or the control group, which got no treatment. Over eight weeks, the experimental group performed daily home myofascial release therapy on their lower limbs using a foam roller. Safety was monitored through weekly phone calls. Secondary outcomes included pain intensity (measured with a visual analog scale), ROM (using a goniometer), functional capacity (assessed with the two-minute walk test), and muscle strength (measured with a dynamometer). Assessments were conducted at the start, at eight weeks, and 10 weeks. The study concluded that myofascial release therapy is a safe physical therapy option for patients with hemophilia. It also found that this therapy, when used alongside prophylactic medications, improved the ROM in dorsal flexion, functional capacity, and gastrocnemius muscle strength [14]. The study by Yin et al. (2022) aims to determine if adding Maitland mobilization to standard rehabilitation exercises is more effective than using standard exercises alone for individuals with chronic ankle instability. The goal is to suggest a new rehabilitation method for this condition. The findings indicate that combining Maitland mobilization with standard rehabilitation exercises can lead to greater improvements in balance and ankle mobility compared to standard exercises alone, although the increase in muscle strength was not significantly different [15].

Ultrasound is a valuable diagnostic and therapeutic tool for both humans and animals due to its non-invasive and repeatable nature. Over the past six decades, acute soft tissue injuries have led to chronic orthopedic and rheumatologic disorders. This research examined the clinical efficacy of therapeutic ultrasound (TUS) in relieving acute and chronic musculoskeletal pain by regulating inflammation and facilitating soft tissue repair. The results indicate that TUS is useful in treating specific musculoskeletal soft tissue pain problems. However, inconsistent findings in research preclude making clear positive recommendations or omitting TUS from clinical practice. TUS has demonstrated significant success in phonophoresis, which includes administering medication by ultrasound, with no documented ill effects. TUS has been widely used to treat various musculoskeletal disorders. Despite these findings, there is a need for better-designed studies to strengthen the evidence base [16].

## Conclusions

This case report emphasizes the value of musculoskeletal physiotherapy. Failure to treat these disorders increases the risk of developing PTAO. Despite advances in treatment procedures and the use of optimal surgery to achieve anatomically precise and stable fracture reduction, the long-term risk of PTAO remains very high. As a result, physiotherapy is an important step toward improvement in terms of pain relief, strength, ROM, gait pattern, and overall quality of life. This case report also underscores the importance of preoperative care to ensure that, post-surgery, the hip and knee ranges are normal and the patient experiences less difficulty walking. Future studies are needed to further investigate this condition and to evaluate the use of ultrasound therapy, which, in our case, was not effective in reducing pain.
